# Responsive evaluation of stakeholder dialogue as a worksite health promotion intervention to contribute to the reduction of SEP related health inequalities: a study protocol

**DOI:** 10.1186/s12913-020-5020-2

**Published:** 2020-03-12

**Authors:** Hanneke van Heijster, Jantien van Berkel, Tineke Abma, Cécile R. L. Boot, Emely de Vet

**Affiliations:** 1 Department of Social Sciences, Chair group Consumption & Healthy Lifestyles, Wageningen University & Research, Hollandseweg 1, 6706 Wageningen, KN The Netherlands; 2 Department of Medical Humanities, Amsterdam UMC, VU University, Amsterdam, The Netherlands; 3 Department of Public and Occupational Health, Amsterdam Public Health research institute, Amsterdam UMC, VU University, Amsterdam, The Netherlands

**Keywords:** Worksite health promotion, Health inequalities, Stakeholder dialogue, Responsive evaluation

## Abstract

**Background:**

Large health inequalities exist in the Netherlands among individuals with a high compared to a low socioeconomic position. Worksite health promotion interventions are considered promising to reduce these inequalities, however, current interventions seem not to have the desired effects. This study proposes ‘moral case deliberation’, a form of stakeholder dialogue on moral dilemmas, as an integrated and inclusive intervention for worksite health promotion. This intervention takes into account three factors that are considered possible underlying causes of low effectiveness of current interventions, namely the lack of deliberate attention to: 1) the diverging values and interests of stakeholders in worksite health promotion, 2) the ethical issues of worksite health promotion, and 3) the connection with the lived experience (lifeworld) of lower SEP employees. Moral case deliberation will help to gain insight in the conflicting values in worksite health promotion, which contributes to the development of a vision for worksite health promotion that is supported by all parties.

**Methods:**

The intervention will be evaluated through Responsive Evaluation, a form of participatory research. Key to Responsive Evaluation is that stakeholders are consulted to determine relevant changes as a result of the intervention. The intervention will be evaluated yearly at both fixed moments (baseline and annual evaluation(s)) and continuously. Mixed methods will be used, including interviews, participatory observations, analyses of HRM-data and short questionnaires. In addition, the intervention will be evaluated economically, on both monetary and non-monetary outcomes.

**Discussion:**

This protocol proposes an innovative intervention and a novel participatory evaluation in the context of worksite health promotion. The study aims to gain understanding in how dialogue on moral dilemmas on health and health promotion can contribute to heightened personal and mutual understanding among stakeholders and practice improvements in the work context. By evaluating the intervention in more than one setting, findings of this study will provide knowledge about how MCD can be adapted to specific work settings and what changes it may lead to in these settings.

**Trial registration:**

Netherlands Trial Register (NRT): NL8051. Registration date: 28/09/2019, retrospectively registered. https://www.trialregister.nl/

## Background

Large health inequalities exist in the Netherlands between individuals with an high socioeconomic position (SEP) and individuals with a low SEP. Low SEP individuals are expected to live 7 years shorter than those with high SEP, and about 19 years shorter in good health [1, 2]. These SEP-related health inequalities can be explained by differences in conditions in the physical environment (e.g. housing and working conditions) and the social environment (e.g. social support), and by behavioural factors (e.g. lifestyle) [3]. Health inequalities are often discussed in terms of injustice: those who are already favoured in wealth also have better chances of being healthy [4]. Individuals with poor health have less opportunities to live their life as they want to, because their health situation may impede social, economic or societal participation. Besides that, poor health is associated with higher societal costs [4], and decreased economic productivity in organizations [5]. In 2014, approximately 20% of Dutch individuals aged 25 and older had a low SEP [6]. Hence, it is warranted from an individual, organisational and societal perspective, to explore ways to promote health among low SEP individuals.

Worksite health promotion (WHP) is considered promising to promote health among lower SEP employees for two reasons. First, because the worksite gives access to the generally hard to reach low SEP population, as half is employed [7] and employees spend much time of their lives at work [8, 9]. Second, because the workplace facilitates an integrated or ‘social ecological’ approach for health promotion by allowing to target a combination of both individual and contextual factors that influence health [10], such as working conditions, social support and lifestyle [3]. Thus, the work setting can be enabling and facilitating for integrated approaches of health promotion to promote health of lower SEP employees.

Yet, current literature gives reasons to doubt on whether WHP interventions in their current form can reduce health inequalities. A meta-analysis [11] showed that WHP interventions that include a cognitive and educational component are more effective in promoting healthy lifestyle for higher SEP employees than for lower SEP employees. Also, a systematic review showed that WHP interventions focusing on health education were ineffective in decreasing socioeconomic health inequalities [12]. Moreover, a summary of literature reviews on the effectiveness of WHP aiming at promoting healthy lifestyles of employees in general (rather than specific lower SEP employees), concludes that WHP interventions have positive, but small effects overall [13]. Consequently, it has been questioned whether these small effects are the result of the intervention itself (theory-failure), or of the way interventions are implemented or evaluated (programme failure) [14]. Therefore, it is warranted to look at the possible underlying causes of the small effects of WHP for employees in general, and for the even smaller effects for lower SEP employees in particular.

A first underlying reason for low effectiveness in general might be the lack of acknowledgement of diverging values and interests of the many stakeholders that are involved in WHP. Stakeholders such as the employer, employees, intervention providers, research and knowledge institutes and insurance companies, all have their own interests regarding WHP [15]. For example, employers may want to promote employees’ health for cost-saving aspects, sustainable employability in the light of aging workforce, and good employment practice for company image building. Intervention providers at their turn want to sell their programs to employers, as that is how they derive their reason of existence [16]. For employees however, it is not self-evident that they receive WHP programs with open arms [17]. Employees go to work to for example earn a living, develop themselves, build on meaningful work relations with colleagues, and/or contribute to something valuable [18], but not necessarily to get their health promoted [19]. Interventions should pay attention to this multiplicity of values and interests at stake.

Following on this is the second possible underlying cause for small effects in general, which is the lack of awareness about the ethical side of WHP. Employees can experience WHP interventions as interference in their privacy, which in its turn can a play role in employees’ decision whether to or not to participate [20]. On the other hand, as employees depend on their employer to maintain their job, employees might feel coerced to participate in WHP interventions [16]. Also, questions such as how far an employer can go in promoting employees’ health often rises [20] as well as whether (and to what extent) employees are responsible for their health or whether their employer is [19]. To conclude, it is important to take into account the ethical questions and the conflicting values that come along with WHP, as they can influence participation in WHP and the relationships at the workplace.

A third possible reason for low effectiveness in general is that employees generally lack voice in WHP [19]. This is considered of particular importance for lower SEP employees because, although there is ample knowledge about the health issues that lower SEP employees generally face (e.g. unhealthier lifestyle or poorer working conditions), insight is scarce in how these people experience their work and health promotion interventions, and how to target their health effectively considering their lifeworld [13]. The possible influence of lifeworld, as conceptualized by Habermas [21], on WHP can be found in a qualitative study among low SEP individuals [22]. These individuals indicated they were aware of the negative consequences of certain health behaviours, yet changing these behaviours had no priority due to other problems in their lives for which they indicated to ‘need’ certain types of unhealthy behaviours. Similar patterns may be seen in WHP: lower SEP employees may find health and health promotion important, yet the specific work setting they are in as well as their personal situation may make them feel powerless or not interested to improve their health.

This project proposes an intervention that takes into account the aforementioned possible underlying causes, which together all add to the complexity of WHP. The intervention consists of stakeholder dialogues on moral dilemmas and underlying values, in which various stakeholders of WHP are invited to discuss health and health promotion. Lower SEP employees play a central role in the dialogues, to make sure that health is being discussed from their lifeworld’s perspective. A form of stakeholder dialogue that allows to take into account the aforementioned ethical dimensions of WHP is moral case deliberation (MCD) [23]. MCD is a form of stakeholder dialogue that originates from philosophy, with a theoretical background in pragmatic-hermeneutical and dialogical ethics [23]. The aim of MCD is to create a moral learning process, by bringing together and confronting diverging perspectives and sorting out underlying values and norms. By creating a moral learning process with various stakeholders about moral issues related to health and health promotion, MCD aims to enhance the personal and mutual understanding and support for WHP, in which moral dilemmas have been taken into account. This may lead to for example improved working relations and mutual understanding among stakeholders on short-term, to enhanced experience of control and autonomy of employees on medium term and to an improved perception of health and well-being on long-term.

The type of evaluation of this project, Responsive Evaluation, will be supportive in adapting MCD to the context of WHP, in which it has not been used before. Stakeholder participation is the starting point of Responsive Evaluation, allowing stakeholders to be consulted about their ideas, needs and wishes regarding the adaptation of MCD to their work setting. Responsive Evaluation is a form of interactive, participatory research, making use of mixed methods [24] and aims to heighten the personal and mutual understanding of multiple stakeholders through dialogue, as the first step towards practice improvement. In Responsive Evaluation stakeholders are involved in the research process by formulating research objectives and relevant changes in consultation with them, and by continuously keeping stakeholders updated about findings during the research process. Additionally, Responsive Evaluation pays attention to silenced groups, such as employees in WHP, facilitating to take the lived experiences of lower SEP employees as the starting point for evaluation. Thus, the intervention and evaluation in this study are not isolated, but complementary and grounded in similar epistemological assumptions on the co-creation of knowledge [25].

## Objectives

1. Develop an integrated worksite health promotion intervention consisting of moral case deliberation to discuss moral issues related to health and health promotion.
2. Evaluate the implementation of and changes due to moral case deliberation in the context of worksite health promotion, and the adaptations needed to make moral case deliberation relevant for the context of work health promotion and its stakeholders.
3. Evaluate the economic impact of moral case deliberation as an intervention for worksite health promotion on economic outcomes, both monetary (Budget Impact Analysis) and non-monetary (Social Return on Investment).

## Methods/design

### Aim 1: Develop an integrated intervention consisting of moral case deliberation (MCD)

#### Dialogue method

To structure a MCD session, a variety of dialogue methods can be chosen [26]. Within this project the ‘Dilemma Method’ will form the basis of the intervention. The method is considered suitable for the work setting, as this method offers the most tangible and down-to-earth approach of the situation, and it is suitable for both life-or-death decisions and everyday issues. The MCD sessions will be guided by trained facilitators (HvH, JvB). The facilitator functions as a non-directive facilitator as opposed to an expert and concentrates on the quality of the deliberation process by guarding the quality of the dialogue [23, 26]. In the MCD sessions, there are six to twelve participants.

#### Continuous adaptations intervention

Responsive Evaluation allows for continuous adaptation of the intervention to the (changing) context and needs for the target population. There are some adaptations that can be expected upfront. Firstly, the level of the language used in the dialogue sessions and other forms of communication will be adapted to match the literacy level of the participants. Abstract use of (ethical) concepts will be avoided and all forms of communication will be adjusted to B1 literacy level. Secondly, to match the work context, the timing of the MCD sessions have to be adapted to be both feasible and relevant for the organisation. The duration of the MCD sessions will therefore be reduced from the duration of 1,5 h (which is common in settings in which MCD is often performed, such as health care, detention, army), to 1 h. Further adaptations will be carried out throughout the evaluation period.

#### Recruitment

The aim is to include a maximum variety of stakeholders in the dialogues in order to capture a broad range of experiences and perspectives. Stakeholders will be contacted by means of a contact person in the participating organisations or directly and selected based on their willingness to participate.

### Aim 2: Evaluate the implementation of and changes due to MCD

#### Study design

A Responsive Evaluation design will be used to evaluate the implementation and the eventual changes due to MCD. As described before, Responsive Evaluation is characterized by stakeholder involvement, yet the degree of involvement may range from collaborative evaluation, participatory evaluation and empowerment evaluation [27]. The evaluation in this project has the ‘lightest’ degree of participation, namely collaborative evaluation. In collaborative evaluation, stakeholders are consulted and involved in the evaluation, but the evaluator remains in mainly in charge of the decisions, thus power.

**Fig. 1:**
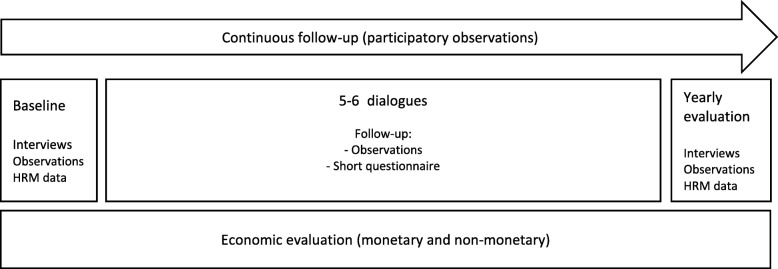
Schematic overview of the Responsive and Economic Evaluation (1 year minimum, 2 years maximum)

The implementation and impact of the intervention will be evaluated both at fixed moments as well as continuously. The fixed moments are at the beginning of the project (baseline) and a yearly evaluation. The minimum duration of the intervention is 1 year, with a maximum of 2 years. The baseline and yearly evaluation comprise mixed methods such as interviews, participatory observations and analyses of HRM-data. Continuous evaluation comprises participatory observations and short questionnaires. Figure 1 provides a schematic overview of the Responsive Evaluation. The overview includes the economic evaluation (aim 3).

#### Study population

The study population consists of lower SEP employees within two Dutch organisations (maximum MBO 1 – comparable to middle school- or lower [6]). The intervention will be implemented in two organisations, namely a harbour service provider, and a sheltered workplace for employees with disabilities. Higher SEP employees are not excluded from the intervention: the intervention is based on the rationale that it should lead to changes in the context. Therefore, the entire all employees of the organisations should be allowed to participate. All employees may benefit from the intervention, however, as health problems are more prevalent for lower SEP employees, they are expected to benefit most from the intervention. Names of the organizations will not be made public, only in case of explicit written consent of each organisation.

#### Data collection

##### Baseline

At baseline, information about relevant changes as a result of the intervention according to the stakeholders, prerequisites for participation and for the adaptation of the MCD sessions to the specific work context, and information about the work setting will be gathered. The three aims will be discussed separately below:

#### Relevant changes as a result of the intervention

Relevant changes are those changes that are considered important from the perspectives of the various stakeholders. For the management and staff, a relevant change might be improved job satisfaction, mutual understanding or general well-being of employees. Some examples of relevant changes for line-managers and employees might be for example learn about how to deal with stressful situation at work, improved communication in the organisation or contribute to a culture in which a healthy lifestyle is promoted.

#### Prerequisites for participation and for adaptation of MCD to the work context

Prerequisites for participation are important, as it is not self-evident for employees to come to the table and talk about health. Also other stakeholders such as the management might have time related restrictions in their possibilities to participate in the dialogues. Prerequisites include suitable timing of the MCD sessions, duration and composition of the group. As described before, the lower SEP employees are the starting point for the question who would they like to be in dialogue with. This will be re-evaluated throughout the intervention period.

#### Insight into the work setting

Insight into the work context (type of work, the working conditions, terminology) of the organisation is necessary for better and correct interpretation of the data. To obtain the information for the baseline, the following methods will be used:

#### Interviews

Interviews will be held with approximately 6 stakeholders. The interviews will be audio recorded and transcribed verbatim.

#### Participatory observations

Job shadowing (i.e. participating in meetings and work routines) will be performed by the researchers, as a means to help interpret interview findings [28]. In addition, participatory observations are a means to build trust in the organisations. This trust is important to create a safe communication climate in the dialogues [24]. The quantity of participatory observations at baseline will depend on the possibilities and flexibility within the organisations. Field notes will be taken during or shortly after the observations. Researchers (HvH and JvB) will discuss their observations afterwards.

#### HRM-data analyses

HRM data will be used to analyse relevant changes that were formulated by stakeholders. These changes will be evaluated yearly. The type of data depends on the changes considered relevant by the various stakeholders, but could for example include indicators such as job satisfaction.

##### Change evaluation

The goal of the change evaluation is to gain insight in perceived changes, personal experiences, and attitudes towards the intervention according to the stakeholders [29]. The methods of that were used at baseline will partly be repeated, including interviews, participatory observations, and HRM-data. Additionally, transcripts of the MCD sessions will be used as input of the further process, subtract feedback, intermediate findings and relevant topics for subsequent MCD sessions.

#### Data analysis

##### Qualitative data

Qualitative data will be analysed through thematic content analysis [30]. For the baseline and MCD data, the data will be the starting point of the analysis instead of a theoretically-driven or predefined coding scheme (inductive approach). Within the inductive approach both semantic and latent strategies will be used. Semantic strategies (descriptive codes)**,** are used to code practical information about the work context and terminology. The latent strategy (codes that require interpretation) will be used to analyse underlying ideas, mechanisms and values.

For the annual evaluation, a deductive inductive approach will be applied. First, a deductive approach will be applied to analyse the data on the existence of changes. The division of levels of aims/outcomes of MCD will be used (case, individual, team and organisational) [31]. Subsequently, an inductive approach will be applied in order to analyse any underlying mechanisms, mechanisms and values in the data.

#### Enhancing quality

To enhance quality, reliability and validity of the qualitative data, several techniques will be taken into account [28]. First, interpretations of interviews will be presented to interviewees to verify correctness *(member check).* Also, multiple data sources (such as interviews and participatory observations) will be used and combined in analyses *(data triangulation).* Third, data will be coded by (at least) two researchers (HvH, JvB) and discussed within the multidisciplinary research team *(investigator triangulation).* Fourth, the researchers (post-doc and PhD) will keep a diary to reflect on their role and influence in the research process *(reflexivity)*. Finally, researchers (HvH and JvB) will document decisions and developments, and the underlying reasons *(audit trail)*.

##### Quantitative data

Change indicators of HRM data are monitored over time, taking into account different organisational levels and subgroups of employees. Therefore, these quantitative data will be analysed according to principles of longitudinal multilevel regression analysis. Change will be determined from the perspective of all employees, but there will be a specific focus on the health related changes among lower SEP employees (subgroup analyses).

### Aim 3: Economic evaluation of the stakeholder dialogue intervention

The intervention will also be evaluated economically, through a Budget Impact Analysis (BIA) and a Social Return on Investment (SROI) analysis.

### Budget impact analysis (BIA)

A BIA is a means to analyse the financial changes after the adoption of a new intervention, by comparing costs before and afterwards the intervention [32]. Examples of costs that can be compared pre and post intervention are productivity related costs or absence and presenteeism related costs. Which costs will be chosen depends on what data is available in the organisation. A possible change in costs will be measured yearly compared to a baseline measure. The eventual change in costs will be compared to the investment made for the intervention. For this project the investment comprises the costs for the MCD sessions, such as costs for the time stakeholders spent on participating in the intervention, for training and implementation of facilitators, and for overhead.

### Social return on investment (SROI)

The non-monetary economic evaluation will be performed through Social Return on Investment (SROI). SROI is a framework for measuring change in social, environmental and economic outcomes that are relevant to stakeholders and uses monetary values to represent them [33]. Data for the SROI will be gathered yearly by interviewing stakeholders that are relevant for the specific context of the participating organization. Stakeholders of SROI are defined as people or organisations that experience change, whether positive or negative, as a result of the activity being analysed [33].

#### Data for SROI

In order to perform a SROI, data needs to be collected on input, output, outcomes, indicators, financial proxies and contribution of the intervention [33]. Input refers to what investment the intervention entails for each stakeholder, such as time or money. Output refers to what is concretely delivered as part of the intervention, such as a certain amount of stakeholder dialogues. The outcomes are the changes perceived by stakeholders, as established in the responsive evaluation. General examples of outcomes given by the SROI Guide (2012) [33] are ‘reduced social isolation’, or an ‘increase in recycling’. Indicators are the concrete expression of the outcome, such as ‘frequency of social contact with friends’ and ‘amount of waste going to landfill’. Once the outcomes and indicators are mapped, financial proxies i.e. monetary values have to be given to the defined outcomes. There are several strategies to find these financial proxies (SROI guide). An example of a strategy is ‘Revealed Preference’, in which financial proxies are defined by inferring valuation from the prices of related market-traded goods. At last, data should be collected on the extent to which the intervention has contributed to the outcomes (attribution, drop-off and displacement). These factors are measured as percentages and are used to make a more accurate estimate of the total value of the outcomes.

### Ethics approval and consent to participate

This study is approved by the Social Ethics Committee, on behalf of Wageningen University and Research. Potential participants are asked to participate in the research via a contact person in the participating organizations. They communicate their decision to the contact person, so consent to participate will be given orally. Before each interview and MCD session, participants will be asked to give written informed consent to record the interviews and MCD sessions. The information will be presented in a level of literacy that is considered acceptable for all employees (B1 literacy level). For observations, there will be an oral informed consent, given by the person in charge of the situation that is being observed. If anyone objects against observations, the researchers will not perform the observation. Both informed consent forms state that data from interviews, MCD sessions and observations will only be accessible for two researchers in this project (HvH, JvB).

## Discussion

This paper describes the study protocol of the development and evaluation of moral case deliberation (MCD) as a worksite health promotion (WHP) intervention. MCD was chosen as a form of stakeholder dialogue because it pays attention to the multiplicity of values and interests in WHP and moral dilemmas at stake, employees’ lived experiences and power asymmetries in the work context. Responsive Evaluation was proposed as the type of evaluation of this study. This study will add to the current body of literature of WHP, as it provides a novel intervention as well as an original type of evaluation in the field of WHP.

The strength of this study is that Responsive Evaluation allows to respond to the dynamic and ambiguous context of the work setting. Organisations are constantly changing due to internal and external developments, which also changes the context in which WHP interventions are being performed. In order to maintain appropriateness of the intervention in a changed situation, Responsive Evaluation as well as the intervention offer the flexibility to adapt research goals and activities during the research process [24]. Ambiguity in the work setting is a result of power relations such as the dependency relationship between employer and employee. These power relations are also reflected in WHP, where the employer is in charge and the employee has little or no voice in WHP. The starting point of Responsive Evaluation is a bases of equality among various stakeholders [24, 34], as well as special attention to silenced voices such as employees in WHP, which makes this type of evaluation very suitable for the work setting.

To date, Responsive Evaluation is considered innovative in the field of WHP, where a Randomized Controlled Trial (RCT) is the gold standard. A RCT aims to evaluate causality by randomizing participants in an intervention and control group, and correcting for bias in the analysis [35]. One important difference between both types of evaluation is that Responsive Evaluation does not aim to evaluate causality. Instead, Responsive Evaluation aims to support the stakeholders in the setting under study to better understand their context themselves, which allows and supports them to make relevant practice improvements.

Responsive Evaluation results in context-specific outcomes. In the field of social sciences and health promotion, there have been discussions about the external validity of such context-specific outcomes [34, 36, 37]. In her commentary, Carminati (2018) [37] proposes transferability as an alternative term for generalizability for research that comprises only or mainly context bound data instead of quantitative data. Transferability means that outcomes of a study can be ‘transferred’ to other contexts by the readers through extrapolation and application of the ‘thick description’ of the findings [24, 37]. In this project, findings will provide organisations and researchers knowledge about how MCD was adapted to two different work settings, and what changes MCD led to in those settings. To ensure quality of transferability in this study several measures will be taken [38], such as discussing findings’ resonance with existing literature from different settings.

In the present study, health inequalities associated with socioeconomic position (SEP) are the starting point of this study. We acknowledge that intersectionality between SEP and other social categories such as gender, age, ethnicity, disability and first language, also influence chances on good health and thus health inequalities, due to various factors [39, 40]. The intersection between social categories should eventually be the starting point of interventions that aim to reduce health inequalities. Although the intervention and evaluation method of this study allows to adapt to the context of an organisation, including its population, it can be questioned whether diversity in all its facets is optimally taken into account, as the main focus is on SEP.

Additionally, WHP interventions alone cannot reduce SEP related health inequalities, as only half of the low SEP population is working [7]. This means that the other half does not have a job and therefore cannot be reached through the work setting. Moreover, these low SEP individuals do not profit from the positive effects of work on health and general well-being [41]. Therefore, these non-employed low SEP individuals presumably deal with poorer health conditions than employed lower SEP employees. Therefore, it is highly recommended to develop health promotion interventions, which may include dialogue methods as well, for non-employed low SEP individuals through other settings.

This protocol describes the development and evaluation of a worksite health promotion intervention consisting of moral case deliberation. The findings of this study may contribute to the body of literature about worksite health promotion and health inequalities, by evaluating moral case deliberation in two different work settings. Also, this study will provide novel insights into the suitability of Responsive Evaluation in worksite health promotion as an alternative to RCT. The results of this study are expected to be available in 2021–2022.

## Data Availability

Not applicable.

## Abbreviations

MCD: Moral case deliberation
RCT: Randomized controlled trial
SEP: Socio-economic position
WHP: Worksite health promotion
